# PI Polynomial of V-Phenylenic Nanotubes and Nanotori

**DOI:** 10.3390/ijms9030229

**Published:** 2008-02-28

**Authors:** Vahid Alamian, Amir Bahrami, Behrooz Edalatzadeh

**Affiliations:** 1 The Organization for Educational Research and Planning (OERP), Iran; 2 Department of Mathematics, Islamic Azad University, Garmsar Branch, Garmsar, Iran; 3 Department of Mathematics and statistics, Shahid Beheshti University, Tehran, Iran; E-mail: b_edalatzadeh@sbu.ac.ir

**Keywords:** PI polynomial, molecular graph, phenylenic nanotube and nanotorus

## Abstract

The PI polynomial of a molecular graph is defined to be the sum X^|E(G)|−N(e)^ + |V(G)|(|V(G)|+1)/2 − |E(G)| over all edges of G, where N(e) is the number of edges parallel to e. In this paper, the PI polynomial of the phenylenic nanotubes and nanotori are computed. Several open questions are also included.

## 1. Introduction

Let *G* be a simple molecular graph without loops, directed and multiple edges. The vertex and edge sets of G are represented by V(*G*) and E(*G*), respectively. A topological index is a numeric quantity derived from the structural graph of a molecule. Usage of topological indices in chemistry began in 1947, when Harold Wiener developed the most widely known topological descriptor, the Wiener index, and used it to determine physical properties of the type of alkanes known as paraffins [1]. The Hosoya polynomial of a graph *G* is defined to be W(*G;X*) = ∑_uv ∈V(_*_G_*_)_(X)^d(^*^u,v^*^)^ where d(*u,v*) denotes the length of a minimum path between u and v. In [2], Hosoya used the name Wiener polynomial while some authors later used the name Hosoya polynomial.

Let *G* be a connected molecular graph and *e=uv* an edge of G, n*_eu_*(*e|G*) denotes the number of edges lying closer to the vertex *u* than the vertex *v*, and n*_ev_*(*e|G*) is the number of edges lying closer to the vertex *v* than the vertex *u*. The Padmakar-Ivan (PI) index of a graph G is defined to be PI (*G*) = ∑*_e_*_∈ E(_*_G_*_)_[n*_eu_*(*e*|*G*) + n*_ev_*(*e*|*G*)], see [3] and [4].

In a series of papers [5, 6] Ashrafi *et al*. defined a new polynomial which they named the Padmakar-Ivan polynomial. They abbreviated this new polynomial as PI(*G,X*), for a molecular graph *G*. We define PI(*G;X*) = ∑*_uv_*_∈ V(_*_G_*_)_ X^N(^*^u,v^*^)^ where for an edge *e* = *uv*, N(*u,v*) = n*_eu_*(*e*|*G*) + n*_ev_*(*e*|*G*) and zero otherwise. This polynomial is very important in computing the PI index. This newly proposed polynomial, PI(*G,X*), does not coincide with the Wiener polynomial (W (*G,X*)) for acyclic molecules.

**Figure 1.**
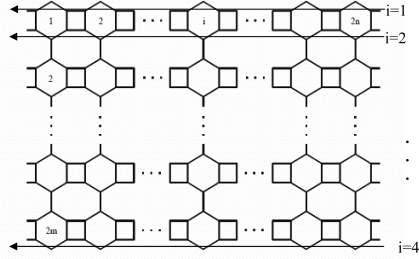
A V-Phenylenic Nanotube.

**Figure 2.**
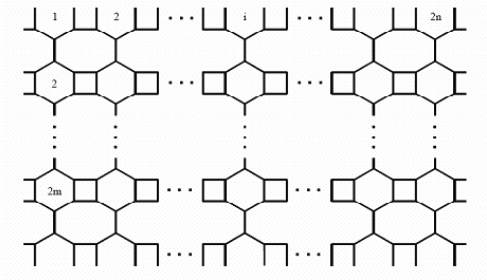
A V-Phenylenic Nanotorus.

In a series of papers [7, 8] Diudea *et al*. investigated the structure and computed the Hosoya polynomial of some nanotubes and nanotori. Gutman *et al*. [9] also computed the Hosoya polynomials of some benzenoid graphs. In [10] Shoujun *et al*. investigated the Hosoya polynomials of armchair open-ended nanotubes. Also, in [5] and [6] the authors computed the PI and Wiener Polynomial of some nanotubes and nanotori. In this paper we continue this program to compute the PI polynomial of V-phenylenic nanotubes and nanotori, using the molecular graphs in Figures 1 and 2. Throughout this paper, the notation is the same as in [11] and [12].

## 2. Results and Discussion

The novel phenylenic and naphthylenic lattices proposed can be contructed from a square net embedded on the toroidal surface. In this section, the PI polynomial of a V-Phenylenic nanotube and nanotorus are computed. Following Diudea [13] we denote a V-Phenylenic nanotube by T=VPHX[4*n*,2*m*]. We also denote a V-Phenylenic nanotorus by H=VPHY[4*n*,2*m*]. Let G be an arbitrary graph. For every edge *e, we* define

$$\text{N}(e)=|\text{E}(\text{G})|-({\text{n}}_{eu}(e|\text{G})+{\text{n}}_{ev}(e|\text{G}))\text{.}$$

By Theorem 1 in [6] we have:

$$\text{PI}(\text{G},\text{X})=\sum_{e\in \text{E}(\text{G})}{\text{X}}^{\left|\text{E}(\text{G})|-N(e\right)}+\left(\begin{array}{c} |\text{V}(\text{G})|+1\\ \text{2} \end{array}\right)-|\text{E}(\text{G})|.$$

So it is enough to compute N(*e*), for every edge *e* ∈ E(G). From above the argument and Figures 1 and 2, it is easy to see that |E(T)|=36*mn*–2*n*, |E(H)|=36*mn* and |V(T)| =24*mn*, |V(H)| =24*mn*. In the following theorem we compute the PI polynomial of *the molecular graphT* in Figure 1.

$$\begin{array}{l} \text{Theorem 1}.\text{ PI(T},\text{X)}={\text{(X}}^{\left(36mn-6n\right)})\;(8mn)+({\text{X}}^{\left(36mn-4n\right)})\;(4mn-2n)+({\text{X}}^{\left(36mn-2n-8m\right)})\;(8mn)\\ +\{\begin{array}{cc} \;\;\;\;\;\;\;\;\;\;\;\;\;\;\;\;\;\;\;\;\;\;\;\;\;\;\;\;\;\;\;\;\;\;\;\;\;\;\;\;\;\;\;\;\;\;\;\;\;\;\;\;\;\;\;\;\;\;\;\;\;\;\;\;\;\;X^{36mn-6n}(16mn) & \;\;\;\;\;,if\;m\leq \frac{m}{2}\\ 2\left(\overset{4m-2n}{\underset{i=1}{\Sigma }}\{2n({\text{X}}^{36mn-6n-2i+2})\}+(n-m)(4n){\text{X}}^{36mn-2n-8m}\right) & if\;m>\frac{n}{2}\text{ and }m<n\\ 2\left(\overset{2n}{\underset{i=1}{\Sigma }}\{2n({\text{X}}^{36mn-6n-2i+2})\}+(m-n)(4n){\text{X}}^{36mn-10n+2}\right) & \;\;\;\;\;\;,m\geq n \end{array}\\ \;\;\;\;\;\;\;\;\;\;\;\;\;\;\;\;\;\;\;\;\;\;\;\;\;\;\;\;\;\;\;\;\;\;\;\;\;\;\;\;\;\;\;\;\;\;\;\;\;\;\;\;\;\;\;\;\;\;\;\;\;\;\;\;\;\;\;\;\;\;\;\;\;\;\;\;\;\;+(24mn+1)(12mn+1)-36mn+2n. \end{array}$$

**Proof:** To compute the PI polynomial of *T*, it is enough to calculate N(*e*). To do this, we consider three cases: that *e* is vertical, horizontal or oblique. If *e* is horizontal. a similar proof as Lemma 1 in [14] shows that N(*e*)=8*m*. Also, if *e* is a vertical edge in one hexagon or octagon then N(*e*) = 4*n*, 2*n*, respectively.

We consider the set A(T) of oblique edges in T. For every *e* in A(T), we have two cases:

**Case 1:**

$$Case\;1:\;m\leq \frac{n}{2}$$

A similar argument as Lemma 2 in [14] gives that N(e)=4*n*.

**Case 2:**

$$Case\;2:\;m>\frac{n}{2}$$

We denote the i^th^ row of oblique edges in A(T) by A_i_ (see Figure 1). It is easy to see that by graph symmetry each element of A_i_ has the same number of parallels. If *e*∈ A_i_ and 1≤i≤2(*m*-|*n-m*|), by computations, we have N(*e*)=4*n*+2i-2, also if 2(*m*-|*n-m*|)+1 ≤ i ≤2*m*, then N(*e*)=8*n*-2. If *m*>*n*, then N(e)=8m. For *n>m* because of symmetry computations are similar to u er part of graph. So we have:

$$\sum_{e\;is\;vertical}{\text{X}}^{\left|E|-N(e\right)}=({\text{X}}^{\left(36mn-6n\right)}\;(8mn)+(X^{\left(36\text{mn}-4\text{n}\right)})\;(4mn-2n).$$

and

$$\sum_{e\;is\;horizontal}{\text{X}}^{\left|E|-N(e\right)}=({\text{X}}^{\left(36mn-2n-8m\right)})\;(8mn).$$

Also:

$$\underset{e\;is\;oblique}{\Sigma }X^{\left|E|-N(e\right)}=\{\begin{array}{cc} {\text{X}}^{36mn-6n}(16mn) & ,if\;m\leq \frac{n}{2}\\ \text{2}\left(\overset{4m-2n}{\underset{i=1}{\Sigma }}\{2n({\text{X}}^{36mn-6n-2i+2})\}+(n-m)(4n){\text{X}}^{36mn-2n-8m}\right) & if\;m>\frac{n}{2}\text{ and }m<n\\ 2\left(\overset{2n}{\underset{i=1}{\Sigma }}\{2n({\text{X}}^{36mn-6n-2i+2})\}+(m-n)(4n){\text{X}}^{\text{36}mn-10n+2}\right) & ,m\geq n \end{array}.$$

Thus:

$$\begin{array}{l} \;\;\;\;\;\;\;\;\;\;\;\;\;\;\;\;\;\;\;\;\;\;\;\;\;\;\;\;\;\;\;\;\;\;\;\;\;\;\;\;\;\;\;\;\;\;\;\;\;\;\;\;\;\;\;\;\;\;\;\;\;\;\;\;\;\;\;\;\;\;\;\;\;\;\;\;\;\;\;\;\;\;\;\;\;\;\;\;\;\;\;\;\;\;\;\;\;\;\text{PI}(\text{T},\text{X})=\underset{e\in \text{E}(\text{T})}{\Sigma }{\text{X}}^{\left|\text{E}(\text{T})-N(e\right)}+\left(\begin{array}{c} |\text{V}(\text{T})|+1\\ \;\;\;\;\;\;\;\;\;\;\;\;\;\;\;\;\;\text{2} \end{array}\right)-|\text{E}(\text{T})|\\ =\underset{e\;is\;horizontal}{\Sigma }{\text{X}}^{\left|E|-N(e\right)}+\underset{eisvertical}{\Sigma }{\text{X}}^{\left|\text{E}(\text{T})-N(e\right)}+\underset{e\;is\;oblique}{\Sigma }{\text{X}}^{\left|E|-N(e\right)}+(|\text{V}(\text{T})|+1)(|\text{V}(\text{T})|+2)/2-|\text{E}(\text{T})|\\ =({\text{X}}^{36mn-6n)}\;(8mn)+({\text{X}}^{\left(36mn-4n\right)})\;(4mn-2n)+({\text{X}}^{\left(36mn-2n-8m\right)})\;(8mn)+\\ +\{\begin{array}{cc} {\text{X}}^{36mn-6n}(16mn) & ,if\;m\leq \frac{m}{2}\\ 2\left(\overset{4m-2n}{\underset{i=1}{\Sigma }}\{2n({\text{X}}^{36mn-6n-2i+2})\}+(n-m)(4n){\text{X}}^{36mn-2n-8m}\right) & if\;m>\frac{n}{2}\text{and}m<n\\ 2\left(\overset{2n}{\underset{i=1}{\Sigma }}\{2n({\text{X}}^{36mn-6n-2i+2})\}+(m-n)(4n){\text{X}}^{36mn-10n+2}\right) & ,m\geq n \end{array}\;.\\ \;\;\;\;\;\;\;\;\;\;\;\;\;\;\;\;\;\;\;\;\;\;\;\;\;\;\;\;\;\;\;\;\;\;\;\;\;\;\;\;\;\;\;\;\;\;\;\;\;\;\;\;\;\;\;\;\;\;\;\;\;\;\;\;\;\;\;\;\;\;\;\;\;\;\;\;\;\;\;\;\;\;\;\;\;\;\;\;\;\;\;\;\;\;\;\;\;\;\;\;\;\;\;\;\;\;\;\;\;\;\;\;\;\;\;+(24mn+1)(12mn+1)-36mn+2n \end{array}$$

Which completes the proof.

In our next theorem we consider a V-Phenylenic nanotorus *H* and calculate its Padmakar-Ivan polynomial, PI(*H,X*), Figure 2.

$$\begin{array}{c} Theorem\;2.\text{ PI(H},\text{ X)}=\underset{e\in \text{E(H)}}{\Sigma }{\text{X}}^{\left|\text{E(T)}|-N(e\right)}+\left(\begin{array}{c} |\text{V(H)}|+1\\ \text{2} \end{array}\right)-|\text{E(H)}|=({\text{X}}^{\left(36mn-8n\right)})\text{ (8}mn)+({\text{X}}^{\text{(36mn}-\text{2n)}})\;(4mn)+\\ ({\text{X}}^{\text{(36}mn-8m)})\text{ (8}mn)+\;(16mn)X^{36mn-6z+2}\;(24mn+1)(12mn+1)-36\text{mn} \end{array}$$

where z=min{m,n}.

**Proof:** To prove the theorem, we apply a similar method as in Theorem 1. It is easily seen that N(*e*)=8n for each vertical edge in hexagons, that is two times more twice the tube case by horizontal symmetry. A vertical edge in an octagon has 2n parallels, as in Theorem 1. Also N(e) =8m for each horizontal edge. Let *z* = min{*m,n*}, for each oblique edge *e* we have N(*e*) = 6*z* − 2. So:

$$\begin{array}{c} \underset{e\;is\;vertical}{\Sigma }X^{\left|E|-N(e\right)}=({\text{X}}^{\left(36mn-8n\right)})\text{ (}8mn)+({\text{X}}^{\text{(36mn}-\text{2n)}})\text{ (4}mn).\\ \underset{e\;is\;horizontal}{\Sigma }X^{\left|E|-N(e\right)}=({\text{X}}^{\left(36mn-8n\right)})\text{ (}8mn).\\ \underset{e\;is\;oblique}{\Sigma }X^{\left|E|-N(e\right)}=(16mn)X^{36mn-6z+2}. \end{array}$$

Thus:

$$\begin{array}{c} \text{PI}(\text{H},\text{X})=\underset{e\in \text{E}(\text{H})}{\Sigma }{\text{X}}^{\left|\text{E}(\text{H})|-N(e\right)}+\left(\begin{array}{c} |\text{V}(\text{H})|+1\\ 2 \end{array}\right)-|\text{E}(\text{H})|=({\text{X}}^{\left(36mn-8n\right)})(8mn)+({\text{X}}^{\left(\text{36mn}-\text{2n}\right)})(4mm)+({\text{X}}^{\left(36mn-8m\right)})\\ (8mn)+(16mn)X^{36mn-6z+2}+(24mn+1)(12mn+1)(12mn+1)-36\text{mn}. \end{array}$$

and this completes the proof.

We conclude our paper with the following open questions:

**Question 1:** Let $F(x)=\sum_{k=0}^{n}{\left(-1\right)}^{k}x^{k}$ be a polynomial of degree *n*. Is there a V-phenylenic nanotube or nanotorus *T* such that PI (T,*x*) = *F*(*x*)?

**Question 2:** Is it true that for every polynomial F(*x*) with positive coefficients and of degree *n*, there exists a V-phenylenic nanotube or nanotorus *T*, such that PI (*T, x*) = F(*x*)?

**Question 3:** What is the relation between the Hosoya polynomial and PI polynomial of a V-phenylenic nanotube or nanotorus?
